# Distinct morphological drivers of jumping and maneuvering performance in gerbils

**DOI:** 10.1242/jeb.250091

**Published:** 2025-02-13

**Authors:** Courtney G. Reed, Sharon M. Swartz, Bethan L. Littleford-Colquhoun, Madeleine Florida, Logan Torres, Thomas J. Roberts, Tyler R. Kartzinel

**Affiliations:** ^1^ Department of Ecology, Evolution, and Organismal Biology, Brown University, Providence, RI 02912, USA; ^2^ Institute at Brown for Environment and Society, Providence, RI 02912, USA

**Keywords:** Biomechanics, Functional morphology, Gerbillinae, Locomotion, Predator escape, Rodentia

## Abstract

Theoretically, animals with longer hindlimbs are better jumpers, while those with shorter hindlimbs are better maneuverers. Yet, experimental evidence of this relationship in mammals is lacking. We compared jump force and maneuverability in a lab population of Mongolian gerbils (*Meriones unguiculatus*). We hypothesized that gerbils with long legs (ankle to knee) and thighs (knee to hip) would produce the greatest jump forces, while gerbils with short legs and thighs would be able to run most rapidly around turns. Consistent with these hypotheses, gerbils with longer legs produced greater jump forces after accounting for sex and body mass: a 1 mm greater leg length provided 1 body weight unit greater jump force on average. Furthermore, gerbils with shorter thighs were more maneuverable: a 1 mm greater thigh length reduced turn speed by 5%. Rather than a trade-off, however, there was no significant correlation between jump force and turn speed. There was also no correlation between jump force and total hindlimb length, and a weak positive correlation between corner-turning speed and total hindlimb length. These experiments revealed how distinct hindlimb segments contributed in different ways to each performance measure: legs to jumping and thighs to maneuvering. Understanding how variations in limb morphology contribute to overall gerbil locomotor performance may have important impacts on fitness in natural habitats.

## Teaser

**Highlighted Article:** Analysis of jump force and maneuverability in gerbils shows distinct lower legs contribute more to jump performance and thighs to maneuvering.

## INTRODUCTION

A primary goal of functional morphology is to identify connections between organismal form and function (Lauder, 1981; Koehl, 1996; Manafzadeh, 2023), which is challenging because traits often perform multiple functions subject to natural selection (Arnold, 1983; Patek et al., 2004; Uiterwaal et al., 2020). A second challenge is that movement is often studied using specialized species that have evolved unique morphology to operate at the upper limits of performance (Patek et al., 2004; Usherwood and Wilson, 2005). However, more generalized species may evolve traits that enable adequate performance for multiple functions rather than masterful performance in just one (Clemente et al., 2013; Maestri et al., 2016). Thus, a major priority in the field of functional morphology is to understand form–function relationships in the traits of generalist species that may experience opposing selection pressures.

Theoretically, limb morphology may impose a biomechanical trade-off between maximizing performance in jumping and maneuverability in quadrupedal mammals. Vertebrates with longer hindlimbs generally produce greater force and work during jumps (Toro et al., 2004; Moreno-Rueda et al., 2020) because longer bones generate force over a longer time period, allowing muscle shortening to occur over a greater distance, thus producing more kinetic energy during takeoff (Alexander, 1995). Moreover, jumpers rely on quick release of stored elastic energy from tendons, muscles and connective tissue, and longer limbs yield greater tissue mass for storage (Aerts, 1998; Roberts, 2016). By contrast, animals with shorter hindlimbs may have increased maneuverability because (i) limbs can drive acceleration and deceleration over shorter distances, enabling them to change direction more rapidly without over-rotating as they turn (Walter and Carrier, 2002; Jindrich et al., 2006) and (ii) they have a lower center of mass to help maintain stability (Dial et al., 2008). Thus, longer hindlimbs generate greater force, but shorter hindlimbs may provide greater control when rapid maneuvers are required. Thus, there may be a trade-off whereby longer hindlimbs yield better jumps and shorter hindlimbs provide enhanced control.

The individual limb segments that contribute to overall hindlimb length may differentially affect locomotive performance (Alexander, 1995; Freymiller et al., 2022). One reason that longer legs yield stronger jumps is positive allometric scaling between limb length and muscle mass. However, the effect of a length increase on jump performance in each hindlimb segment differs as a result of non-linear scaling of muscle mass, with greater positive allometry for proximal muscles (hip and knee extensors) compared with distal muscles (ankle extensors; Freymiller et al., 2022). Thus, individual hindlimb segments may differ in the degree of their impact on jump performance, which cannot be captured by total limb length alone. Furthermore, maneuverability may also be impacted by individual hindlimb segment lengths. For example, shorter hindlimbs overall are correlated with better corner-turning performance in quadrupedal mammals (Walter and Carrier, 2002; Jindrich et al., 2006; Haagen Sen et al., 2022). However, research in dogs shows that longer tibial segments reduce over-rotation during high-speed turns, and while whole forelimb length is positively associated with turn speed, the strongest effect is associated with the radial segment (Haagensen et al., 2022). Thus, there is a need to be more attentive to how the sums of individual hindlimb segments contribute to the performance of the whole.

Research on locomotive performance in mammals tends to focus on species with highly specialized morphology that yields extreme performance results, such as sprinting in greyhounds (Usherwood and Wilson, 2005) and jumping in bushbabies (Aerts, 1998), jerboas (Moore et al., 2017) and kangaroo rats (Schwaner et al., 2018). However, most rodents are quadrupeds that experience multiple types of selection pressure on their limb morphology, meaning they must perform adequately in several locomotive responses but not necessarily be specialized for any given one of them. Generalist versus specialist traits in rodents have been well studied for traits such as diet and cranial morphology (Maestri et al., 2016) but surprisingly not for rodent locomotion (but see Hedrick et al., 2020). Thus, understanding how morphology affects multiple axes of performance in generalist species is essential for the field of ecomorphology.

We investigated associations between limb morphology and jumping and maneuvering performance for Mongolian gerbils (*Meriones unguiculatus*), quadrupedal rodents that occur naturally across a range of East Asian grasslands and deserts. Based on the theorized trade-off between how limb segments contribute to jumping and maneuvering, we focused on leg and thigh lengths (i.e. knee-to-ankle tibia and knee-to-hip femur, respectively). We hypothesized that (i) gerbils with longer legs and/or thighs would produce greater jump force and (ii) gerbils with shorter legs and/or thighs would round corners faster. We expected that individual limb segments lengths would be more informative than total hindlimb length. We further hypothesized that (iii) the best jumpers to be the worst maneuverers and vice versa.

## MATERIALS AND METHODS

### Study system

We obtained 30 Mongolian gerbils, *Meriones unguiculatus* (Milne-Edwards, 1867) (aged 11–13 weeks; even sex ratio), from Charles River Laboratories. Gerbils were housed singly, given plastic huts, kept on a 12 h light:dark cycle at 24°C, fed ∼4 g per day (T.2016.15; Teklad Lab Animal Diets, Indianapolis, IN, USA), and acclimated for 10 days. All protocols were approved by Brown University's IACUC.

Our primary goal was to evaluate the theoretical link between hindlimb morphology and performance, but we also accounted for traits that are at least occasionally implicated as determinants of jumping and/or maneuvering performance in mammals, such as tail length (e.g. jumping: Carrizo et al., 2014; maneuvering: Libby et al., 2012) and forearm length (maneuvering: Jindrich et al., 2006). For limb measurements, we held gerbils in a one-handed scruff so that we could accurately and repeatably measure them (Machholz et al., 2012). We measured the following traits for each gerbil: left leg (ankle to knee), thigh (knee to hip), hindfoot (heel to toe), forefoot (wrist to fingertip), arm (wrist to elbow) and tail (base to tip) lengths with calipers (±1 mm) and mass with a scale (±0.1 g).

We attempted to weigh and measure animals on each trial date and usually succeeded (mean±s.d. 1±3 days from trial date). Overall, gerbils grew during the experiment, and males were significantly larger than females on average (males 69 g at start, growing 23%; females 62 g at start, growing 9%; Fig. S1). In some cases, we could not measure gerbils that exhibited stress responses such as appearing frozen or excessively excited when touched. To employ appropriate morphological values in our statistical analyses while accounting for growth, we excluded performance trials for which we did not obtain a measurement within ±14 days. We thus had to drop 2 jumping trials and 3 maneuvering trials because we could not obtain measurements within ±14 days of the event. All statistical analyses were run in R version 4.2.1 (http://www.R-project.org/).

### Jumping experiments

To test jump performance, we built a vertical tower (10×10×47 cm) using clear 1/16 mm polycarbonate sheets, including a ∼0.5 cm gap at the bottom (Fig. S2A). The tunnel was designed to exert no weight on the ATI mini40 six-axis load cell and attached force plate at its base. We placed gerbils in the tunnel to acclimate for 30 s and set the force plate to record data at 1000 samples per second using Igor (v7, Wavemetrics, Lake Oswego, OR, USA). Each trial began when gerbils stood on four feet and were sprayed with compressed air to simulate surprise by ambush predators (Moore et al., 2017). We recorded videos at 700 frames s^−1^ using two Phantom MIRO cameras and Phantom Camera Control software. To assess peak jump performance, we completed 2–4 trials for 20 gerbils (*N*=12 males, 8 females); we stopped after 1 trial for 8 gerbils that exhibited stress (2 males, 6 females). We scored each of 57 trials involving 28 individuals as: (i) a ‘jump’ in which the gerbil pushed off the ground vertically using its hindfeet, (ii) a ‘hop’ on all four feet or (iii) any ‘other’ response. We obtained time-matched morphological measurements for 15/17 of the gerbils that jumped (11 males, 4 females; Table 1). We calculated maximum resultant force from the vector sum of forces in the *x*, *y* and *z* directions and expressed this value in proportion to the gerbil's mass. We low-pass filtered force plate data at 70 Hz to account for vibrations. Resultant force outputs were given in body weight units (BWU); a BWU of 1 is equivalent to a jump force of 1× body weight. We used the maximum force recorded for each gerbil for analysis.

**Table 1.** Sample sizes of gerbil trait measures associated with each jumping and maneuvering experiment

Trait	Jumping	Maneuvering		
		135 deg	90 deg	45 deg
Hindfoot	10	22	15	14
Leg	14	22	15	14
Thigh	15	22	14	14
Forefoot	14	12	8	7
Arm	15	17	12	9
Tail	15	12	8	7

The number of top-performance trials per experiment with time-matched trait measurements is shown for each trait.

We tested the first hypothesis that there would be a positive correlation between jump force and leg/thigh length. Using jump force as the response variable, we ran separate ANCOVA with a given trait length as the predictor variable and sex as the covariate, allowing different intercepts between the sexes. Because sex was not significant in either analysis, we simplified to linear models that included segment length with no difference in intercept between the sexes. To investigate relationships between jump force and other relevant traits, we used hindfoot, forefoot, arm, tail and total hindlimb (leg plus thigh) lengths as predictor variables in separate ANCOVA. All analyses accounted for body mass because jump force was expressed in BWU.

### Maneuverability experiments

To quantify maneuverability, we tested corner-turning ability (Wynn et al., 2015). We built polycarbonate corridors of increasingly difficult turn angles (135, 90, 45 deg) with a 120 cm runway into the turn and 80-grip black traction tape to line floors and visually indicate turns (Fig. S2B–D). Gerbils acclimated by exploring the corner and back at least 3 times before we sprayed them with compressed air to begin the trial. We recorded runs at 240 frames s^−1^ using GoPro Hero 10 Black cameras (one above the arena; one to the side showing the full length of the runway). We tested each gerbil up to 3 times on the three angles, allowing >48 h between tests and randomizing the order of angles. We categorized each of 226 trials across 29 individuals (14 male, 15 female) as: (i) a ‘turn’ if the gerbil's body was parallel to the slow-down lane after the turn (47.8%), (ii) ‘no turn’ if it stopped before its body was parallel (50.9%) or (iii) ‘fail’ if the gerbil ran into a wall (1.3%). We excluded failed trials from analyses, along with 15 trials for which a camera malfunctioned and 3 for which we did not have timely trait measurements. The final dataset included 192 trials across 28 individuals (63 for 135 deg, 65 for 90 deg, 64 for 45 deg). To confirm sharper turns were more difficult, we tested for a correlation between the probability of a turn and turn angle using binomial regression in *lme4* (Bates et al., 2015). The model included turn angle, sex and the turn angle×sex interaction as main effects with gerbil identity as a random effect to account for repeated measurements. We also used binomial regression to test for a significant difference in the probability of turning based on morphology. For each trait, we ran a model with trait, angle and sex as main effects and gerbil identity as a random effect.

To test our second hypothesis that gerbils with shorter legs and/or thighs would round corners more rapidly, we calculated turn speed for each maneuverability trial and used this value in a model selection procedure. We tracked the tip of each gerbil's nose through the trial using DLTdv8 (Hedrick, 2008) in MATLAB R2022a (MathWorks, Inc, 1996) to calculate instantaneous velocity using *trajr* (McLean and Skowron Volponi, 2018; https://CRAN.R-project.org/package=trajr) and used filter order 3 with a filter length of 21 to smooth the trajectory. We calculated the average speed around the corner over 0.1 s intervals. The trial with the fastest average speed around the turn was selected for each gerbil and angle, yielding 52 top-performance trials across 24 individuals (11 males, 13 females; Table 1). The model-selection procedure compared 8 generalized linear mixed models for each hindlimb trait: (i) one null model indicating no difference in slope or intercept after accounting for individual identity, and seven biomechanical models: (ii) trait+angle, (iii) trait+sex, (iv) angle+sex, (v) trait+angle+sex, (vi) trait+sex+trait×sex interaction, (vii) trait+angle+trait×angle interaction and (viii) angle+sex+angle×sex interaction. All models included gerbil identity as a random effect to account for multiple angles per individual. We compared models using corrected Akaike's information criterion (AICc) to identify top-ranked models with AICc cumulative weight <0.95 and ΔAICc <4; we concluded there was no significant correlation if the null model ranked among the top models (Burnham and Anderson, 2002). If more than one biomechanical model fell within the top-ranked set, we performed model averaging in *MuMIn* (version 1.47.1, https://CRAN.R-project.org/package=MuMIn). Finally, to investigate other potentially relevant traits, we repeated model-selection procedures for hindfoot, forefoot, arm, tail and total hindlimb lengths. We focused on sex instead of body mass because preliminary analyses revealed it to be a better predictor of maneuvering performance, but we also present body mass results for completeness (Supplementary Materials and Methods and Table S3).

### Jumping–maneuvering relationship

Thirteen gerbils (10 males, 3 females) performed at least one jump and turned the corner at a minimum of one angle (*N*=31 total trials). We ran linear models at each turn angle to test for a correlation between jump force and turn speed. To determine model power to detect changes in turn speed, we performed a power analysis using the powerSim function in *simr* (*N*=100 simulations; Green and MacLeod, 2016; https://CRAN.R-project.org/package=simr).

## RESULTS

### Jumping

**Fig. 1.**
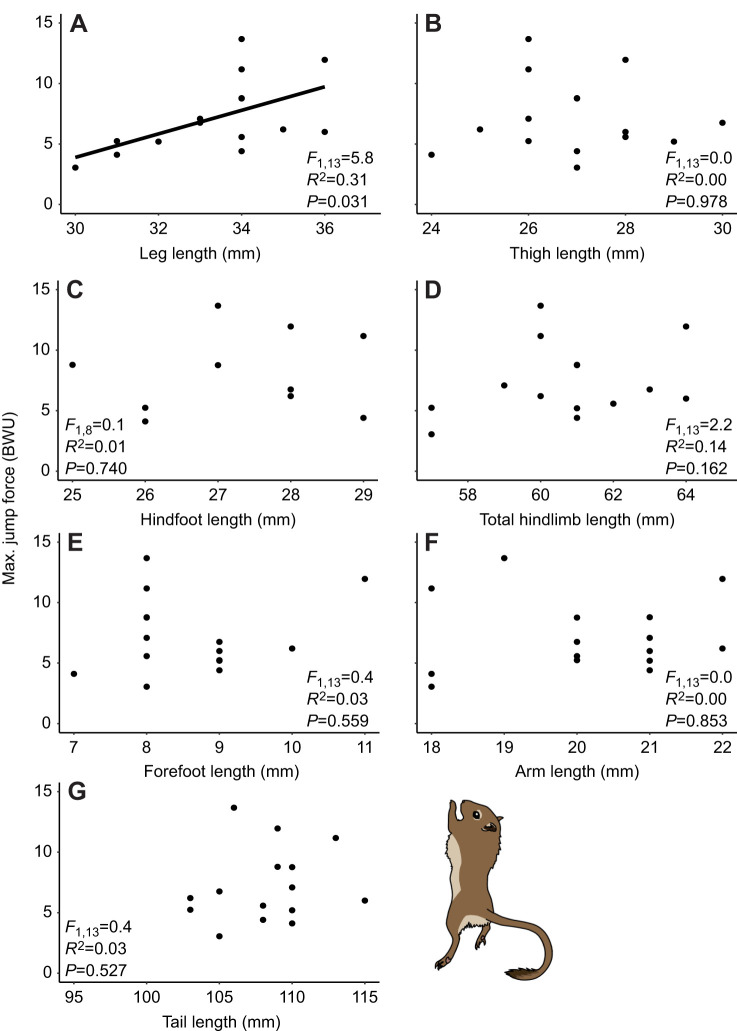
**Relationships between gerbil hindlimb morphology and jump performance.** Jump force increased significantly with leg length (A) but did not differ significantly with thigh (B), hindfoot (C), total hindlimb (D), forefoot (E), arm (F) or tail length (G). Jump force on the *y*-axis is shown in body weight units (BWU) to account for body mass; 1 BWU indicates that the gerbil produced a jump force equivalent to 1× its body weight. Sample sizes are given in Table 1. A and B show results from hypothesis testing; C–G show the results of *post hoc* tests involving additional traits.

There was a significant positive correlation between jump force and body mass (Fig. S3), indicating that larger gerbils produced greater jump force. After accounting for body mass via conversion of force from Newtons to BWU, the results were consistent with our first hypothesis: there was a significant positive correlation between jump force and leg length (Fig. 1A). A 1 mm greater leg length translated to a 1 BWU increase in jump force on average. However, there was not a significant correlation between jump force and thigh, hindfoot, total hindlimb, forefoot, arm and tail length (Fig. 1B–G).

### Maneuvering

**Fig. 2.**
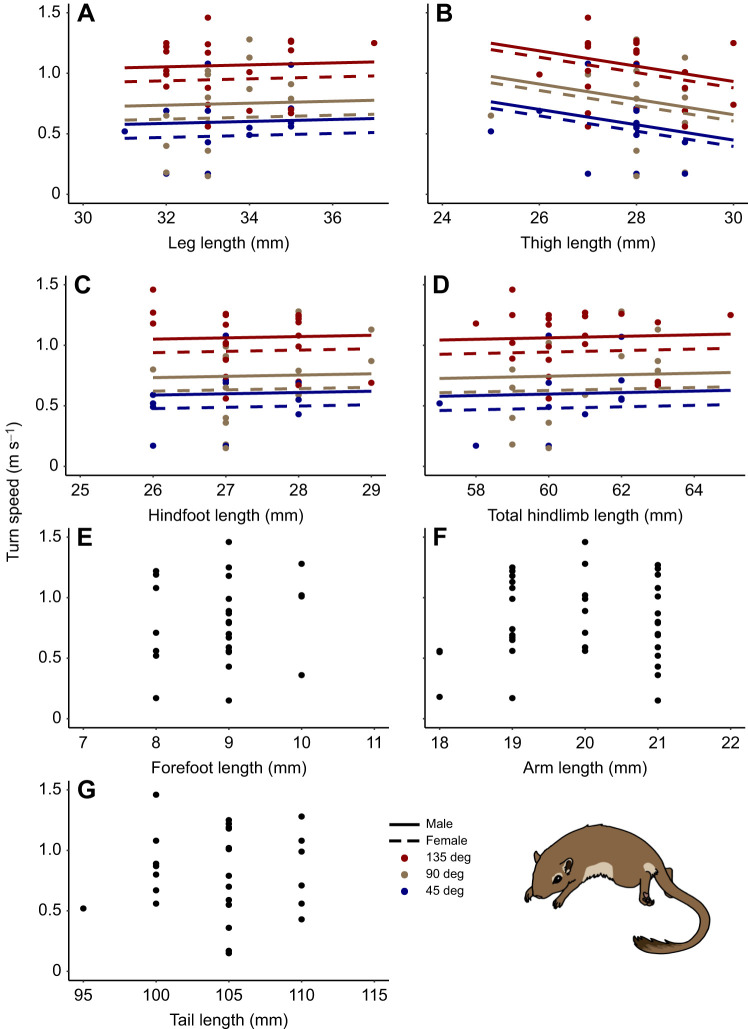
**Relationships between gerbil hindlimb morphology and maneuvering performance.** Turn speed was positively correlated with leg length (A) and negatively correlated with thigh length (B). Turn speed was also slightly positively correlated with hindfoot (C) and total hindlimb length (D). There was no significant association between turn speed and forefoot (E), arm (F) or tail length (G). On average, gerbils ran faster around wider angles, and male gerbils turned faster than female gerbils. Sample sizes are given in Table 1. A and B show results from hypothesis testing; C–G show the results of *post hoc* tests of additional traits. Plotted lines for turn speed were predicted by the average of the top-performing models (Table S2).

The maneuverability experiment challenged gerbils with turns of varying difficulty. The probability of turning increased significantly with wider (easier) angles. There was no significant effect of sex or angle×sex interaction (Fig. S4). There was also no significant difference in turn probability with any of the morphological traits (Table S1). Partially consistent with our second hypothesis, turn speed decreased significantly with thigh length: a 1 mm greater thigh length resulted in a 5% slower turn speed on average. Turn speed was weakly positively correlated with leg length (Fig. 2A) and strongly negatively correlated with thigh length (Fig. 2B). Turn speed was also weakly positively correlated with hindfoot (Fig. 2C) and total hindlimb (Fig. 2D) length. The top models for hindfoot, leg, thigh and total hindlimb length were (i) angle+sex and (ii) trait+angle, with males turning corners faster than females. The null model was ranked in the top set for forefoot, arm and tail lengths (Fig. 2E–G), indicating no significant effect on turn speed. Thus, turn speed was generally faster for wider turns and male gerbils, but speed was modulated such that shorter thighs yielded better performance (Table S2).

### Jumping–maneuvering relationship

**Fig. 3.**
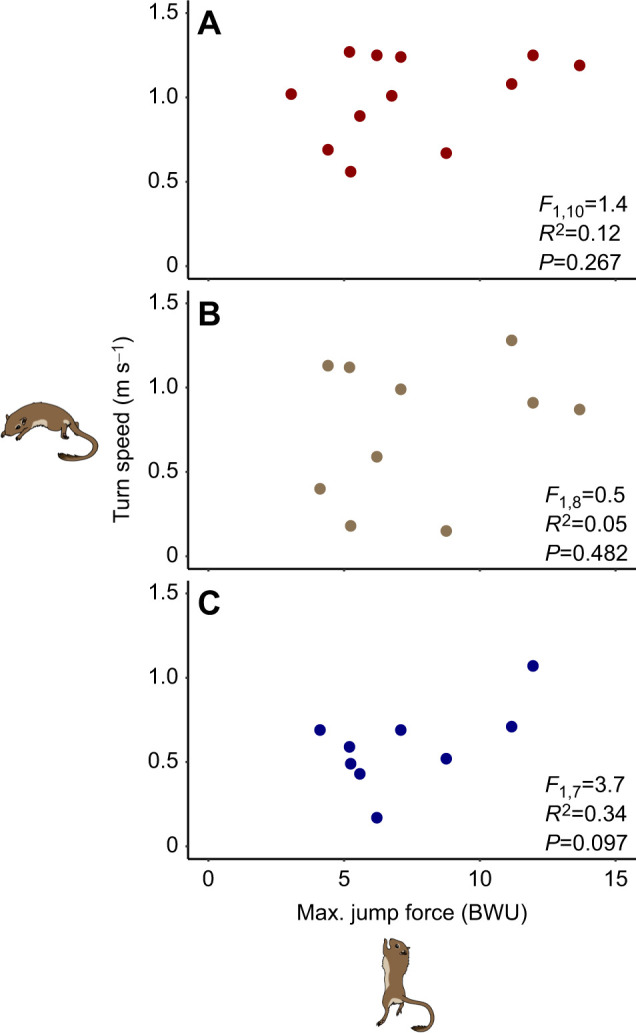
**Lack of correlation between jump force and turn speed.** Models evaluating the correlation between jump force and turn speed at 135 deg (A), 90 deg (B) and 45 deg (C) turn angle yielded no significant correlation. Jump force is shown in BWU; 1 BWU indicates that the gerbil produced a jump force equivalent to 1× its body weight. Sample sizes are given in Table 1.

Contrary to our third hypothesis, there was no negative correlation between jumping and maneuvering performance. There was no significant interaction between jump force and sex at any angle, indicating that the best jumpers were not the worst maneuverers (135 deg: *F*_1,10_=1.4, *R*^2^=0.12, *P=*0.267; 90 deg: *F*_1,8_=0.5, *R*^2^=0.05, *P=*0.482; 45 deg: *F*_1,7_=3.7, *R*^2^=0.34, *P=*0.097; Fig. 3). The power analysis indicated that we had 31% power to detect a 0.03 m s^−1^ change in turn speed.

### Repeatability of jumping and maneuvering results

Of the gerbils that jumped at least once, only 6/17 (35%) performed repeat jumps, and the second-highest jump force was on average 20% lower than the highest. However, every gerbil that performed multiple jumps performed greater than the mean jump force (4.94 BWU) in all of their trials, indicating that any one trial chosen for these gerbils (not just the maximum performance) would still categorize them as above-average jumpers. Of gerbils that turned a corner at least once at a given angle, 32/52 (62%) turned the corner at least twice. Of those that ran at least twice at a given angle, the top two attempts were within an average of 16% of each other.

## DISCUSSION

We tested for a biomechanical trade-off between jumping and maneuvering performance based on limb morphology in gerbils. Consistent with our hypotheses, we found that hindlimb segments influenced performance in distinct ways: gerbils with longer legs were better at jumping (Fig. 1A), while those with shorter thighs were better at maneuvering (Fig. 2B). Moreover, the contributions of individual segment lengths were not captured by the results of total hindlimb (Figs 1D and 2D). Surprisingly, instead of the predicted jumping versus maneuvering trade-off (Toro et al., 2004; Clemente and Wilson, 2015), we found no correlation between jumping and maneuvering performance. We found that jumping and maneuvering performance were relatively consistent around the median trait values but were more variable in gerbils with either very short or very long limb segments (Figs 1 and 2). This consistency, along with the high degree of repeatability across trials for individual gerbils, provides confidence in our results and indicates that the impact of morphology on performance may be more important at extreme values. Although there has been a strong focus on biomechanical relationships between hindlimb length and jump force (Aerts, 1998; Roberts, 2016; Moreno-Rueda et al., 2020), ours is among the first studies to evaluate how hindlimb length variation may jointly contribute to both jumping and maneuvering performance in the same animals.

If different traits confer advantages in jumping (longer legs) and maneuvering (shorter thighs), it raises the question of why populations might maintain variation in these traits. We can identify at least three non-exclusive possibilities. First, limb morphology may be involved with other important behaviors, such as burrowing (Biknevicius, 1993) and climbing (Clemente et al., 2013), which may exert different selection pressures on hindlimbs. Second, hindlimb length–function relationships operate in the context of the rest of the organism's body. Thus, performance may also be contingent on variation in length ratios between forelimbs and hindlimbs, the muscle mass of limbs and/or the way in which elastic energy is generated through the interactions of muscles, tendons, bones and connective tissues throughout the body (Roberts, 2016). Third, small mammals with body plans that are not specialized for a particular style of either jumping or maneuvering may engage in a complex combination of both behaviors in ecologically relevant scenarios. For example, jerboas (family Dipodidae) combine jumping with unpredictable movement trajectories in the air as a means of escaping predators (Moore et al., 2017). During our study, we observed a behavioral response in 15/16 trials: gerbils hopped from all four legs onto their hindlimbs before jumping with their hindfeet. This postural reset enabled countermovement jumps whereby an animal squats from a standing position, extending the muscles before contraction and thus increasing elastic energy (McErlain-Naylor et al., 2014; van Hooren and Zolotarjova, 2017) and jump force (Cavanagh and Komi, 1979; Seiberl et al., 2015). Because movement in ecological contexts (e.g. burrowing, climbing, predator-escape) is often complex, future work to characterize multi-step behaviors may yield insights that extrapolating from independent form–function assays cannot.

Further research within and across species, life stages and experimental designs is needed to identify the range of natural conditions and where our performance results may generally apply. We designed no-choice experimental assays that required individuals to engage in a particular movement associated with predator avoidance, but it can be challenging to obtain and verify maximal performance data in the lab (Astley et al., 2013). In nature, animals would face a choice about whether to jump vertically, run away or exhibit a more complex response (e.g. jumping horizontally). Expanding from analyses involving an outbred lab colony to include morphologically variable wild animals would also help account for the influence of individual-level variation in athleticism or motivation among inexperienced laboratory animals (Wilson et al., 2014). Studies in wild populations would also help account for other relevant sources of variation in natural populations, such as body condition (Almbro and Kullberg, 2008; Rew-Duffy et al., 2020), ontogeny (Stiller and McBrayer, 2013) and sexual dimorphism (Bildstein et al., 1989). For example, male gerbils turned corners faster than females in our study, which is surprising given that the male gerbils are on average larger than female gerbils, and larger mammals typically turn more slowly because of the stronger effects of force limitation (Iriarte-Díaz, 2002; Dick and Clemente, 2017) and positive scaling between rotational inertia and body mass (Carrier et al., 2001; Shankar, 2014). This sex-based difference in performance may instead be related to factors such as sex differences in relative muscle proportions that could be elucidated with future research.

In conclusion, our experiment integrated concepts from biomechanics, evolution and ecology to investigate the form–function relationship between hindlimb morphology and predator-escape performance. We demonstrated that intraspecific trait variation is associated with jumping and maneuvering performance in ways that contradict an expected trade-off between the two strategies. This surprising result highlights the complexity of form–function relationships in species with body plans that are not obviously specialized for either jumping or maneuvering. Because of the diversity of small mammal species that match this description, and the functional importance of small mammals to food webs and ecosystem functioning, determining whether complex trait combinations are associated with locomotive performance within and among species is an exciting research direction at the nexus of biomechanics and ecology.

## Supplementary Material

10.1242/jexbio.250091_sup1 Supplementary information
