# Supplementary material for: Distinct morphological drivers of jumping and maneuvering performance in gerbils
Source: J Exp Biol. 2025 Feb 13;228(3):JEB250091. doi: 10.1242/jeb.250091 (PMC11883271; doi:10.1242/jeb.250091)
Supplement: Supplementary information [file jexbio-228-250091-s1.pdf]

Supplementary Materials and Methods

Model selection for sex vs. body mass

We lacked the sample size to include both sex and body mass in the same models, using the rule of thumb that there should be no more than one term for every ten samples in a model set (Burnham & Anderson, 2002). However, there was a strong association between body size and sex in this population of gerbils, suggesting a degree of redundancy between them (Figure S1). We therefore analyzed the data using sex in the models, as described in the main text. We then analyzed the data using models that included body mass instead of sex. For the set of models including sex, we found that sex consistently appeared as a parameter in our top models for each trait, with male gerbils turning corners faster than female gerbils (Table S2). For the set of models including body mass, we found that body mass only appeared in the top set of models for one of the seven traits (Table S3). The lack of correlation between body mass and turning speed, together with a significant correlation between sex and turning speed, suggests that sex affects performance in a manner that is not simply due to body size differences but may instead be due to sex-specific differences in other factors (e.g., body composition).

References

1. Burnham, K. P. & Anderson, D. R. (2002). Model Selection and Multimodel Inference. Springer New York, NY. http://doi.org/10.1007/b97636

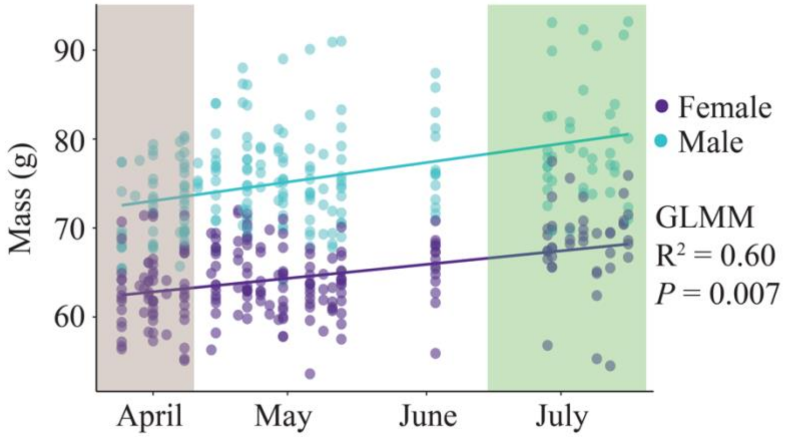

Fig. S1. Change in gerbil body mass over time**.** Gerbils were weighed weekly from March 25 – May 13, 2022, as well as every day during which they ran experimental trials, ending July 16, 2022. Jumping trials were run from March 28 – April 14, 2022, represented by the brown block. Maneuverability trials were run from June 28 – July 16, 2022, represented by the green block. Male gerbils were significantly larger than female gerbils, and gerbils of both sexes increased in mass over the course of the experiments. Mass of male gerbils increased by a mean of 23% (range 6-26%), and female gerbils increased by a mean of 9% (range -5-16%). Trend lines are from a generalized linear mixed model with sex, date, and the sex × date interaction as main effects and gerbil identity as a random effect to account for repeat measurements over time.

Journal of Experimental Biology • Supplementary information

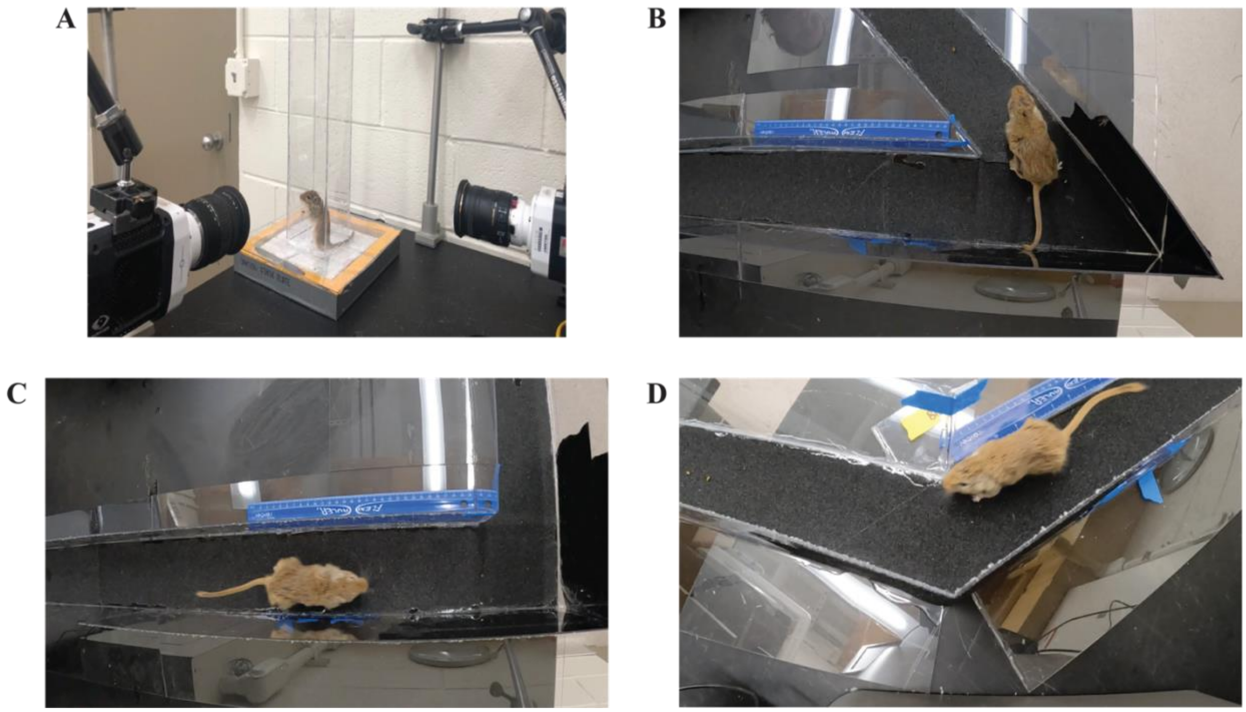

Fig. S2. Experimental set-up for jumping and maneuvering experiments. (A) For jumping assays, gerbils jumped vertically on a force plate in front of two high-speed camera. For maneuvering assays, gerbils ran around corners of increasing difficulty: (B) 45°, (C) 90°, and (D) 135°.

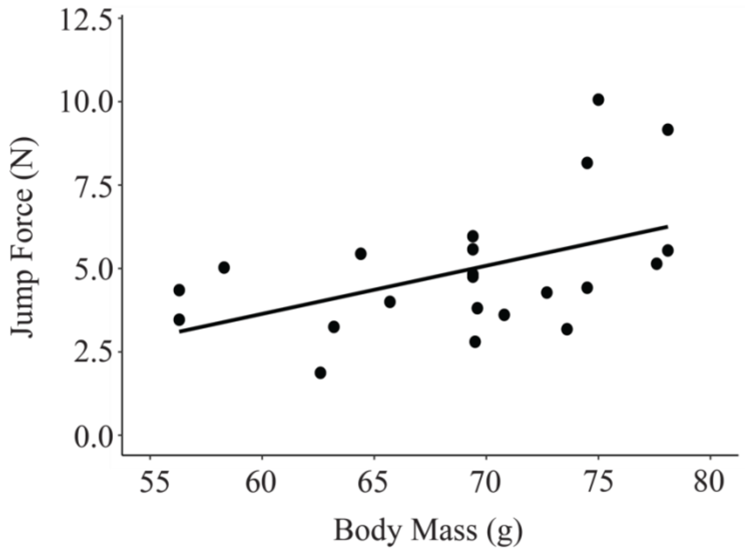

Fig. S3. Body mass and jump force were positively correlated, indicating that larger gerbils produced greater jump force than smaller gerbils.

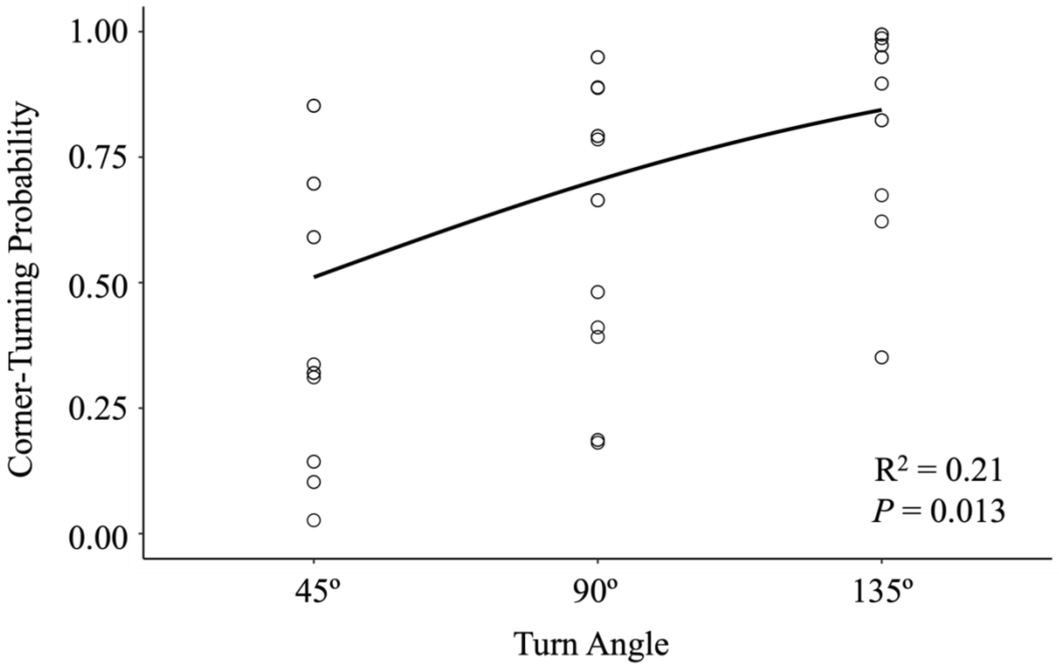

Fig. S4. Model predictions of binomial regression showing that the probability of a gerbil turning a corner increased significantly as the turn angle grew wider and therefore easier (N = 24 gerbils across 51 trials.

Journal of Experimental Biology • Supplementary information

Table S1. Results of binomial regression testing for relationship between morphological traits and probability of a successful corner turn. The same model was run for each trait (trait + angle + sex), and all models included gerbil identity as a random effect to account for repeat measurements. The 'Model' column lists the trait tested in that model. Coefficients (± standard error) are shown for each predictor and model, except for the model for forefoot length, which failed to converge. Parameter significance is indicated as follows: $P < 0.05$*, $P < 0.01$**, and $P < 0.001$***.

	**Coefficients ± *SE***		
	**Trait**	**Angle**	**Sex**
Hindfoot Length	-0.71 ± 0.54	1.96 ± 0.72**	0.32 ± 1.0
Leg Length	-0.73 ± 044	1.98 ± 0.77**	0.71 ± 1.13
Thigh Length	-0.45 ± 0.39	1.89 ± 0.01**	0.08 ± 0.98
Forefoot Length	NA	NA	NA
Arm Length	0.77 ± 1.01	3.20 ± 1.59*	-0.75 ± 2.0
Tail Length	-0.15 ± 0.14	2.91 ± 1.48*	-0.71 ± 1.49
Body Mass	0.05 ± 0.08	1.78 ± 0.67**	-0.73 ± 1.31

Journal of Experimental Biology • Supplementary information

Table S2. Generalized linear mixed model results for turn speed. The same 8 models were run for each of the morphological variables, and all models included gerbil identity as a random effect to account for repeat measurements. The 'Model' column lists the parameters in each model. AICc is Akaike's Information Criterion (AICc). ΔAICc is the difference in AICc between each model and the best-fit model for that trait. The 'Cum.Wt' column gives the cumulative sum of Akaike's weights and reflects the likelihood that the models to that point are the best in the set. Models up to a ΔAICc value of 4.00 and a cumulative weight of 0.95 are considered roughly equivalent in fit. R^2 represents the proportion of variance explained by each model. Coefficients (± standard error) are shown for each predictor and model. Rows are organized in blocks according to each morphological trait. Within those blocks, models are listed in decreasing order of support according to ΔAICc. When multiple models fell within a cumulative weight < 0.95 and a ΔAICc < 4, we performed model averaging and reported the results in the first row of that block.

**Model**					**Coefficients ± *SE***					
	**AICc**	**ΔAICc**	**Cum.Wt**	**R^2**	**Trait**	**Angle**	**Sex**	**Trait × Angle**	**Trait × Sex**	**Angle × Sex**
**Leg**										
Model-Averaged Results					0.01 ± 0.02	0.33 ± 0.06	0.12 ± 0.10			
Angle + Sex	39.2	0.0	0.76	0.35		0.33 ± 0.06	0.14 ± 0.09			
Leg + Angle	42.1	2.9	0.94	0.34	0.04 ± 0.03	0.32 ± 0.06				
Leg + Angle + Sex	46.2	7.0	0.96	0.36	0.03 ± 0.04	0.33 ± 0.06	0.12 ± 0.10			
Angle × Sex	46.3	7.1	0.98	0.37		0.34 ± 0.09	0.17 ± 0.10			-0.04 ± 0.12
Null	47.3	8.1	1.00	0.00						
Leg × Angle	49.7	10.5	1.00	0.41	0.08 ± 0.04	2.27 ± 1.57		-0.06 ± 0.05		
Leg + Sex	57.1	17.9	1.00	0.04	0.05 ± 0.04		0.04 ± 0.11			
Leg × Sex	62.2	23.0	1.00	0.05	0.01 ± 0.08		-1.98 ± 3.09		0.06 ± 0.09	
**Thigh**										

Model-Averaged Results					-0.06 ± 0.04	0.34 ± 0.06	0.05 ± 0.10			
Angle + Sex	37.7	0.0	0.63	0.36		0.34 ± 0.06	0.16 ± 0.09			
Thigh + Angle	39.7	2.1	0.86	0.35	-0.06 ± 0.04	0.34 ± 0.06				
Thigh + Angle + Sex	41.6	3.9	0.95	0.39	-0.07 ± 0.04	0.35 ± 0.06	0.19 ± 0.10			
Angle × Sex	43.2	5.6	0.99	0.41		0.34 ± 0.08	0.19 ± 0.10			-0.02 ± 0.12
Null	46.2	8.5	1.00	0.00						
Thigh × Angle	52.0	14.4	1.00	0.34	-0.06 ± 0.04	0.53 ± 1.68		-0.01 ± 0.06		
Thigh + Sex	56.2	18.5	1.00	0.04	-0.04 ± 0.05		0.13 ± 0.11			
Thigh × Sex	61.4	23.8	1.00	0.04	-0.05 ± 0.06		-0.57 ± 2.89		0.02 ± 0.10	
**Hindfoot**										
Model-Averaged Results					0.01 ± 0.03	0.33 ± 0.06	0.11 ± 0.10			
Angle + Sex	39.2	0.0	0.72	0.35		0.33 ± 0.06	0.14 ± 0.09			
Hindfoot + Angle	41.8	2.6	0.92	0.33	0.05 ± 0.05	0.31 ± 0.06				
Hindfoot + Angle + Sex	45.7	6.4	0.95	0.35	0.02 ± 0.06	0.32 ± 0.06	0.13 ± 0.10			
Angle × Sex	46.3	7.1	0.97	0.37		0.34 ± 0.09	0.17 ± 0.10			-0.04 ± 0.12
Hindfoot × Angle	46.6	7.4	0.99	0.39	0.07 ± 0.05	3.17 ± 2.21				
Null	47.3	8.1	1.00	0.00						
Hindfoot + Sex	55.9	16.7	1.00	0.05	0.09 ± 0.07		0.04 ± 0.11			
Hindfoot × Sex	60.6	21.4	1.00	0.05	0.10 ± 0.11		0.52 ± 3.67		-0.02 ± 0.13	
**Forefoot**										
Null	28.8	0.0	0.80	0.00						
Angle + Sex	32.8	4.0	0.90	0.33		0.33 ± 0.08	0.06 ± 0.14			
Forefoot + Angle	33.6	4.8	0.97	0.34	0.04 ± 0.09	0.31 ± 0.09				
Angle × Sex	36.7	7.9	0.99	0.45		0.25 ± 0.10	0.10 ± 0.14			0.13 ± 0.14
Forefoot + Angle + Sex	39.2	10.4	0.99	0.33	0.05 ± 0.09	0.31 ± 0.09	0.06 ± 0.14			
Forefoot + Sex	39.2	10.4	1.00	0.01	0.04 ± 0.10		0.01 ± 0.15			
Forefoot × Sex	42.6	13.8	1.00	0.05	-0.08 ± 0.15		-2.14 ± 1.83		0.24 ± 0.21	
Forefoot × Angle	43.7	14.8	1.00	0.33	0.05 ± 0.11	-0.33 ± 1.52		0.08 ± 0.18		
**Arm**										

Journal of Experimental Biology • Supplementary information

Null	34.8	0.0	0.39	0.00						
Angle + Sex	32.1	0.3	0.72	0.32		0.32 ± 0.07	0.17 ± 0.11			
Angle × Sex	36.1	1.2	0.93	0.44		0.20 ± 0.10	0.16 ± 0.11			0.17 ± 0.13
Arm + Angle	38.8	3.9	0.99	0.28	0.02 ± 0.05	0.31 ± 0.07				
Arm + Angle + Sex	42.1	7.2	1.00	0.31	-0.02 ± 0.05	0.32 ± 0.07	0.18 ± 0.13			
Arm + Sex	45.2	10.4	1.00	0.03	0.00 ± 0.00		0.12 ± 0.13			
Arm × Sex	50.0	15.2	1.00	0.04	-0.03 ± 0.08		-1.25 ± 2.59		0.07 ± 0.13	
Arm × Angle	50.7	15.9	1.00	0.28	0.02 ± 0.05	-0.20 ± 1.56		0.03 ± 0.08		
**Tail**										
Null	28.8	0.0	0.86	0.00						
Angle + Sex	32.8	4.0	0.97	0.33		0.33 ± 0.08	0.06 ± 0.14			
Angle × Sex	36.7	7.9	0.99	0.45		0.25 ± 0.10	0.10 ± 0.14			-0.45 ± 0.15
Tail + Angle	37.5	8.6	1.00	0.33	0.00 ± 0.02		0.32 ± 0.08			
Tail + Sex	42.7	13.9	1.00	0.00	0.00 ± 0.02		0.01 ± 0.16			
Tail + Angle + Sex	43.0	14.1	1.00	0.32	0.00 ± 0.02	0.33 ± 0.09	0.07 ± 0.15			
Tail × Sex	50.5	21.6	1.00	0.01	0.01 ± 0.03		1.82 ± 4.26		-0.02 ± 0.04	
Tail × Angle	53.8	25.0	1.00	0.36	0.01 ± 0.02	-1.86 ± 2.01		0.02 ± 0.02		
**Total Hindlimb**										
Model-Averaged Results					0.04 ± 0.03	0.33 ± 0.06	0.14 ± 0.09			
Angle + Sex	39.2	0.0	0.77	0.35		0.33 ± 0.06	0.14 ± 0.09			
Trait + Angle	42.3	3.1	0.94	0.34	0.04 ± 0.03	0.31 ± 0.06				
Angle × Sex	46.3	7.1	0.96	0.37		0.34 ± 0.09	0.17 ± 0.10			-0.04 ± 0.12
Trait + Angle + Sex	46.6	7.4	0.98	0.36	0.02 ± 0.03	0.32 ± 0.06	0.11 ± 0.10			
Null	47.3	8.1	0.99	0.00						
Trait × Angle	48.4	9.2	1.00	0.43	0.06 ± 0.3	4.03 ± 2.22		-0.06 ± 0.04		
Trait + Sex	56.5	17.3	1.00	0.07	0.05 ± 0.03		0.01 ± 0.11			
Trait × Sex	62.2	23.0	1.00	0.07	0.03 ± 0.06		-2.3 ± 4.3		0.04 ± 0.07	

Journal of Experimental Biology • Supplementary information

Table S3. Generalized linear mixed model results for turn speed, using body mass as a predictor instead of sex (Table S4). The same 8 models were run for each of the morphological variables, and all models included gerbil identity as a random effect to account for repeat measurements. The 'Model' column lists the parameters in each model. AICc is Akaike's Information Criterion (AICc). ΔAICc is the difference in AICc between each model and the best-fit model for that trait. The 'Cum.Wt' column gives the cumulative sum of Akaike's weights and reflects the likelihood that the models to that point are the best in the set. Models up to a ΔAICc value of 4.00 and a cumulative weight of 0.95 are considered roughly equivalent in fit. R^2 represents the proportion of variance explained by each model. Coefficients (± standard error) are shown for each predictor and model. Rows are organized in blocks according to each morphological trait. Within those blocks, models are listed in decreasing order of support according to ΔAICc. When multiple models fell within a cumulative weight < 0.95 and a ΔAICc < 4, we performed model averaging and reported the results in the first row of that block.

**Model**					**Coefficients ± *SE***					
	**AICc**	**ΔAICc**	**Cum.Wt**	**R^2**	**Trait**	**Angle**	**Mass**	**Trait × Angle**	**Trait × Mass**	**Angle × Mass**
**Leg**										
Trait + Angle	42.1	0.0	0.81	0.34	0.04 ± 0.03	0.32 ± 0.06				
Angle + Mass	46.2	4.1	0.92	0.32		0.32 ± 0.06	0.00 ± 0.01			
Null	47.3	5.2	0.98	0.00						
Trait × Angle	49.7	7.6	1.00	0.41	0.08 ± 0.04	2.27 ± 1.57		-0.06 ± 0.05		
Trait + Angle + Mass	52.7	10.5	1.00	0.34	0.04 ± 0.04	0.32 ± 0.06	0.00 ± 0.01			
Trait + Mass	62.3	20.2	1.00	0.04	0.06 ± 0.05		-0.00 ± 0.01			
Angle × Mass	64.6	22.4	1.00	0.34		0.58 ± 0.70	0.01 ± 0.01			-0.00 ± 0.01
Trait × Mass	71.9	29.8	1.00	0.07	-0.43 ± 0.39		-0.23 ± 0.18		0.01 ± 0.01	
**Thigh**										

Journal of Experimental Biology • Supplementary information

Trait + Angle	39.7	0.0	0.90	0.35	-0.06 ± 0.04	0.34 ± 0.06				
Angle + Mass	45.3	5.6	0.95	0.33		0.33 ± 0.06	0.00 ± 0.01			
Null	46.2	6.5	0.99	0.00						
Trait + Angle + Mass	49.1	9.4	1.00	0.36	-0.07 ± 0.04	0.34 ± 0.06	0.01 ± 0.01			
Trait × Angle	52.0	12.3	1.00	0.34	-0.06 ± 0.04	0.53 ± 1.68		-0.01 ± 0.06		
Trait + Mass	62.1	22.4	1.00	0.02	-0.04 ± 0.05		0.01 ± 0.01			
Angle × Mass	63.9	24.2	1.00	0.34		0.62 ± 0.69	0.01 ± 0.01			-0.00 ± 0.01
Trait × Mass	72.0	32.3	1.00	0.04	-0.50 ± 0.51		-0.17 ± 0.20		0.01 ± 0.01	
**Hindfoot**										
Trait + Angle	41.8	0.0	0.79	0.33	0.05 ± 0.05	0.31 ± 0.06				
Angle + Mass	46.2	4.4	0.87	0.32		0.32 ± 0.06	0.00 ± 0.01			
Trait × Angle	46.6	4.8	0.95	0.39	0.07 ± 0.05	3.17 ± 2.21		-0.10 ± 0.08		
Null	47.3	5.5	1.00	0.00						
Trait + Angle + Mass	52.3	10.5	1.00	0.33	0.04 ± 0.06	0.31 ± 0.06	0.00 ± 0.01			
Trait + Mass	61.3	19.5	1.00	0.05	0.10 ± 0.06		0.00 ± 0.01			
Angle × Mass	64.6	22.8	1.00	0.34		0.58 ± 0.70	0.01 ± 0.01			-0.00 ± 0.01
Trait × Mass	70.5	28.7	1.00	0.06	-0.55 ± 0.82		-0.24 ± 0.30		0.01 ± 0.01	
**Forefoot**										
Null	28.8	0.0	0.91	0.00						
Trait + Angle	33.6	4.8	0.99	0.34	0.04 ± 0.09	0.31 ± 0.09				
Angle + Mass	38.2	9.4	1.00	0.33		0.32 ± 0.08	-0.00 ± 0.01			
Trait × Angle	43.7	14.8	1.00	0.33	0.05 ± 0.11	-0.33 ± 1.52		0.17 ± 0.22		
Trait + Mass	44.2	15.4	1.00	0.01	0.04 ± 0.11		-0.00 ± 0.01			
Trait + Angle + Mass	44.5	15.7	1.00	0.33	0.05 ± 0.10	0.31 ± 0.09	-0.00 ± 0.01			
Angle × Mass	52.3	23.5	1.00	0.43		-0.45 ± 0.91	0.01 ± 0.01			0.01 ± 0.01
Trait × Mass	53.8	25.0	1.00	0.02	-0.64 ± 1.04		-0.09 ± 0.13		0.00 ± 0.01	
**Arm**										
Null	34.8	0.0	0.86	0.00						
Trait + Angle	38.8	3.9	0.98	0.28	0.02 ± 0.05	0.31 ± 0.07				
Angle + Mass	42.2	7.3	1.00	0.28		0.31 ± 0.07	0.00 ± 0.01			

Journal of Experimental Biology • Supplementary information

Trait + Angle + Mass	49.1	14.3	1.00	0.27	0.02 ± 0.05	0.31 ± 0.07	0.00 ± 0.01			
Trait × Angle	50.7	15.9	1.00	0.28	0.02 ± 0.05	-0.20 ± 1.56		0.03 ± 0.08		
Trait + Mass	50.9	16.1	1.00	0.01	0.02 ± 0.06		0.00 ± 0.01			
Angle × Mass	51.7	16.8	1.00	0.40		-0.37 ± 0.84	0.01 ± 0.01			0.01 ± 0.01
Trait × Mass	59.8	25.0	1.00	0.04	-0.83 ± 0.81		-0.23 ± 0.22		0.01 ± 0.01	
**Tail**										
Null	28.8	0.0	0.98	0.00						
Trait + Angle	37.5	8.6	0.99	0.33	0.00 ± 0.02	0.32 ± 0.08				
Angle + Mass	38.2	9.4	1.00	0.33		0.32 ± 0.08	0.00 ± 0.01			
Trait + Mass	47.8	18.9	1.00	0.00	0.00 ± 0.00		0.00 ± 0.00			
Trait + Angle + Mass	48.3	19.5	1.00	0.32	0.00 ± 0.02	0.32 ± 0.09	-0.00 ± 0.01			
Angle × Mass	52.3	23.5	1.00	0.43		-0.45 ± 0.91	0.01 ± 0.01			0.01 ± 0.01
Trait × Angle	53.8	25.0	1.00	0.36	0.01 ± 0.02	-1.86 ± 2.01		0.02 ± 0.02		
Trait × Mass	60.3	31.5	1.00	0.00	0.06 ± 0.29		0.09 ± 0.42		0.00 ± 0.00	
**Total Hindlimb**										
Model-Averaged Results					0.04 ± 0.03	0.31 ± 0.06	0.00 ± 0.01			
Trait + Angle	42.3	0.0	0.78	0.34	0.04 ± 0.03	0.31 ± 0.06				
Angle + Mass	46.2	3.9	0.89	0.32		0.32 ± 0.06	0.00 ± 0.01			
Null	47.3	5.0	0.96	0.00						
Trait × Angle	48.4	6.1	1.00	0.43	0.06 ± 0.03	4.03 ± 2.22		-0.06 ± 0.04		
Trait + Angle + Mass	52.9	10.6	1.00	0.34	0.03 ± 0.03	0.31 ± 0.06	0.00 ± 0.01			
Trait + Mass	61.3	19.0	1.00	0.07	0.07 ± 0.04		-0.00 ± 0.01			
Angle × Mass	64.6	22.2	1.00	0.34		0.58 ± 0.70	0.01 ± 0.01			0.00 ± 0.01
Trait × Mass	71.0	28.7	1.00	0.11	-0.38 ± 0.31		-0.37 ± 0.26		0.01 ± 0.00	

Journal of Experimental Biology • Supplementary information
